# Activin A Protects Midbrain Neurons in the 6-Hydroxydopamine Mouse Model of Parkinson’s Disease

**DOI:** 10.1371/journal.pone.0124325

**Published:** 2015-04-22

**Authors:** Sandy Stayte, Peggy Rentsch, Kong M. Li, Bryce Vissel

**Affiliations:** 1 Neuroscience Department, Garvan Institute of Medical Research, Sydney, New South Wales, Australia; 2 Faculty of Medicine, UNSW Australia, Sydney, Australia; 3 School of Biotechnology and Biomolecular Sciences, UNSW Australia, Sydney, Australia; 4 Pharmacology Department, Bosch Institute, Sydney Medical School, The University of Sydney, Sydney, Australia; Prince Henry's Institute, AUSTRALIA

## Abstract

Parkinson’s disease (PD) is a chronic neurodegenerative disease characterized by a significant loss of dopaminergic neurons within the substantia nigra pars compacta (SNpc) and a subsequent loss of dopamine (DA) within the striatum. Despite advances in the development of pharmacological therapies that are effective at alleviating the symptoms of PD, the search for therapeutic treatments that halt or slow the underlying nigral degeneration remains a particular challenge. Activin A, a member of the transforming growth factor β superfamily, has been shown to play a role in the neuroprotection of midbrain neurons against 6-hydroxydopamine (6-OHDA) *in vitro*, suggesting that activin A may offer similar neuroprotective effects in *in vivo* models of PD. Using robust stereological methods, we found that intrastriatal injections of 6-OHDA results in a significant loss of both TH positive and NeuN positive populations in the SNpc at 1, 2, and 3 weeks post-lesioning in drug naïve mice. Exogenous application of activin A for 7 days, beginning the day prior to 6-OHDA administration, resulted in a significant survival of both dopaminergic and total neuron numbers in the SNpc against 6-OHDA-induced toxicity. However, we found no corresponding protection of striatal DA or dopamine transporter (DAT) expression levels in animals receiving activin A compared to vehicle controls. These results provide the first evidence that activin A exerts potent neuroprotection in a mouse model of PD, however this neuroprotection may be localized to the midbrain.

## Introduction

Parkinson’s disease (PD) is a chronic neurodegenerative disease characterized by significant loss of dopaminergic neurons within the substantia nigra pars compacta (SNpc) and a subsequent loss of dopamine (DA) within the striatum. While the use of levodopa (L-Dopa) has provided considerable benefits in the treatment of the motor symptoms, one of the remaining challenges in PD research is the search for therapeutic treatments that address the underlying nigral degeneration. One of the most promising avenues in recent years has been the administration of growth factors to promote the survival of remaining midbrain neurons and stimulate the growth of process from immature neurons.

While their exact mechanisms of action still remain to be fully elucidated, growth factors have been demonstrated to act as neuroprotective molecules against cytotoxic cell damage via upregulation of calcium buffering proteins, antioxidant enzymes, and anti-apoptotic factors [1] suggesting that their administration either prior to, or after a neurotoxic insult may provide protection to vulnerable neuronal populations. These properties make growth factors ideal as therapeutic targets as PD.

Activin A is a member of the transforming growth factor (TGF)-β superfamily, which are known to be involved in development, inflammation and repair of tissues and organs. A number of studies have shown that activin A may play a particular role in the neuroprotection of CNS regions and functions following injury. *In situ* hybridization studies have demonstrated an increase in activin A mRNA expression in neurons near the site of CNS injury following lesioning with the excitotoxin kainic acid (KA) [2] as well as increased expression up to 7 days after unilateral hypoxic-ischemic injury in the rat brain [3].

The neuroprotective potential of activin A was first investigated in the KA model of acute brain injury, with the administration of exogenous recombinant activin A exerting potent neuroprotection against KA-induced excitotoxicity in the hippocampus [4]. Interestingly, further investigations revealed that in the presence of the activin-binding protein follistatin, the neuroprotective action of fibroblast growth factor 2 (FGF-2; a well characterized neuroprotective molecule) was lost in the KA model, suggesting that activin A may be an essential mediator for neuroprotection in degenerative models [4].

In addition to its known neuroprotective effects in the hippocampus, activin A has been shown to exert neuroprotective effects in midbrain neurons *in vitro*, with the addition of recombinant activin A to dopaminergic neurons isolated from rat mesencephalon floor resulting in more tyrosine hydroxylase (TH) positive cells after 8 days in culture compared to untreated controls, representing a 2.5 fold increase in survival of dopaminergic neurons. Furthermore, the presence of activin A significantly reduced MPP^+^-induced cell loss when administered prior to exposure with the neurotoxin [5]. More recently, addition of activin A to human SH-SY5Y neuroblastoma cells attenuated both degeneration induced by serum withdrawal and 6-OHDA administration, with this neuroprotection inhibited by follistatin [6]. The ability of activin A to provide significant neuroprotection *in vitro* against established toxins used to model the pathological events of human PD in animals, suggests that activin A may provide a novel target for halting midbrain degeneration. Our study provides the first evidence that exogenous application of activin A prior to 6-OHDA administration provides significant protection of midbrain neurons from degeneration *in vivo*.

## Materials and Methods

### Animals

C57BL/6 male mice aged 12 weeks were obtained from Australian BioResources (Mona Vale, Australia). Mice were housed at a maximum five mice per cage, until the study began, at which time mice were housed individually. Mice were kept on a 12-hour light/dark cycle with access to food and water *ad libitum*. All animal experiments were performed with the approval of the Garvan Institute and St. Vincent’s Hospital Animal Ethics Committee under approval number 12/36, in accordance with National Health and Medical Research Council animal experimentation guidelines and the Australian Code of Practice for the Care and Use of Animals for Scientific Purposes (2004). All surgery was performed under ketamine/xylazil anesthesia, and all efforts were made to minimize suffering. For all immunohistochemical experiments animals were anaesthetized via a ketamine/xylazil mixture before cardiac perfusion with 4% paraformaldehyde. For all rapid euthanasia and tissue collection animals were anaesthetized via isofluorane followed by cervical dislocation.

### Osmotic micro-pump implantation

For all neuroprotection studies animals were anaesthetized with a mixture of ketamine (8.7 mg/ml; Mavlab, Slack Creek, QLD) and xylazil (2 mg/ml; Troy Laboratories Pty Ltd, Smithfield, Australia) and placed in a stereotaxic apparatus (Kopf Instruments, Tujunga, California) on the day prior to lesioning. Osmotic micro-pumps (Model 1007D; Alzet, Palo Alto, California) were filled with 24.5 ng/μl (294 ng total daily dose) of recombinant human/mouse/rat activin A (R&D Systems) or vehicle control (1x PBS) and implanted subcutaneously along the back of the neck as previously described [7]. An infusion cannula (PlasticsOne, Roanoke, US) connected to the micro-pump was placed in the left lateral ventricle (to ensure the cannula did not impede subsequent lesioning) at AP -0.26, ML -1.0, DV -2.8 relative to bregma. The pump was removed 1 week later and the cannula tubing sealed with heat.

### Unilateral striatal lesioning

Animals were anaesthetized with a mixture of ketamine (8.7 mg/ml; Mavlab, Slack Creek, QLD) and xylazil (2 mg/ml; Troy Laboratories Pty Ltd, Smithfield, Australia) and placed in a stereotaxic apparatus (Kopf Instruments, Tujunga, California). Mice were then injected with 2 x 2 μl of 3 mg/mL (total 12 μg) of 6-hydroxydopamine (6-OHDA; Sigma Aldrich, Australia) in 0.02% ascorbic acid (sham) in the right sensorimotor striatum at the following coordinates: AP +1.0, ML +2.1, DV -2.9 and AP +0.3, ML +2.3, DV -2.9, relative to bregma and the dural surface as previously described [8]. 6-OHDA (or 0.02% ascorbic acid control) was injected at a rate of 0.5 μl/minute and the syringe left in place for 2 minutes after each injection to allow for complete diffusion.

### Immunohistochemistry

Mice were anaesthetized with ketamine (8.7 mg/ml; Mavlab, Slack Creek, QLD) and xylazil (2 mg/ml; Troy Laboratories Pty Ltd, Smithfield, Australia) and transcardially perfused with ice-cold phosphate-buffered saline (PBS) and 4% paraformaldehyde (PFA) 1, 2, or 3 weeks after lesioning. For all neuroprotection studies animals were transcardially perfused 3 weeks after lesioning. Brains were harvested and postfixed in 4% PFA at 4°C overnight and then cryoprotected in 30% sucrose. Tissue was cryosectioned at 40 μm and stored in PBS with 0.02% sodium azide at 4°C until used. For all sections endogenous peroxidases were quenched using 3% H_2_O_2_ in 50% ethanol for 20 minutes and were subjected to blocking with 3% Bovine Serum Albumin (BSA) + 0.25% Triton in 1x PBS for 1 hour at room temperature to block all non-specific sites. Sections were then incubated in the following primary antibodies: monoclonal mouse tyrosine hydroxylase (TH, 1:1000 Sigma Aldrich, Australia cat # T2928), monoclonal mouse neuronal nuclei (NeuN 1:500, Merck Millipore, Australia cat # MAB377), monoclonal rat dopamine transporter (DAT 1:1000, Merck Millipore, Australia cat # MAB369) for 72 hours at 4°C. All sections were then incubated in the respective biotin-labeled secondary antibodies (1:250, Sapphire Bioscience cat # AB6813, Life Technologies, Australia cat # B-2770, Sigma Aldrich, Australia cat # B7139) overnight at 4°C followed by incubation in avidin-biotin complex (Vector Laboratories, USA) at room temperature for 1 hr. TH and DAT immunolabeling was detected with 3,3’-Diaminobenzidine (DAB, Abacus) until desired staining achieved. NeuN immunolabeling was detected with DAB intensified with nickel ammonium sulfate and counterstained with polyclonal rabbit anti-TH (1:1000, Merck Millipore cat # AB152) that was detected with Nova-Red (Abacus) to outline the substantia nigra region.

### Stereology

Quantification of SNpc cell population estimates was performed using the optical fractionator method and the use of Stereo Investigator 7 software (MBF Bioscience). For the estimations of TH positive populations a counting frame of 60 μm x 60 μm and a grid size of 75 μm x 85 μm was used. For the estimations of NeuN positive populations a counting frame of 65 μm x 65 μm and a grid size of 135 μm x 145 μm was used. For all cell types the guard zone height used was 5 μm and dissector height used was 10 μm, with every third section sampled to a total of 10 sections. Coefficient of error attributable to the sampling was calculated according to Gundersen and Jensen [9]. Errors ≤0.10 were regarded as acceptable. The medial lemniscus was used to define the border between the SNpc and VTA and the SNpc was delineated from -2.8 to -3.88 mm relative to bregma based on the Paxinos atlas for the mouse brain [10].

### Catecholamine analysis

Animals were sacrificed by cervical dislocation 3 weeks after lesioning, the brains removed and the striatum rapidly dissected out and snap frozen. The day prior to catecholamine analysis, the mobile phase solution consisting of 0.1 mol/L phosphate buffer (pH 3.0), 0.74 mmol/L PIC B-8 octane sulphonic acid (Waters, Australia), 0.3 mmol/L sodium EDTA, and methanol (18% v/v) was prepared, run through the system, and maintained at a flow rate of 0.7 mL/min overnight. Striata were homogenized with a 1 ml solution of ice-cold 0.2M perchloric acid containing 0.1% L-cysteine and 400 nmol/L of 5-hydroxy-N-nethyltryptamine (5-HMeT) as internal standard. Following homogenization, 50 μl of homogenate was aliquoted for protein analysis and placed on dry ice. The remainder of the homogenate was centrifuged at 15,000g at 4°C for 15 min and 10 μl aliquot of the supernatant was analyzed by HPLC. The HPLC system consisted of a Shimadzu ADVP module (Kyoto, Japan) equipped with a SIL-10 autoinjector with sample cooler and an LC-10 on-line vacuum degassing solvent delivery unit. Chromatographic control, data collection and processing were carried out using Shimadzu Class VP data software. Dopamine (DA), 3,4-dihydroxyphenylacetic acid (DOPAC), homovanillic acid (HVA), and 5-HMeT were separated by a Phenomenex Synergi Polar-RP 80A reversed phase column with Optiguard C18 (1 mm). Quantification was achieved via an intro electrochemical detector (Antec Leyden, Netherlands) equipped with a glassy carbon working electrode set at +0.75V. The calibration curve of each standard was obtained by the concentration versus the area ratio of the standard and internal standard (5-HMeT). Catecholamine levels were standardized to protein levels measured via Bradford assay and recorded as ng/μg of protein. All standards for the Bradford assay were prepared in freshly made 0.2M PCA + 0.1% L-cysteine.

### Densitometry

Optical densitometry was performed on DAT-positive fibres in the striatum using Image Pro Plus 6.0 software (Media Cybernetics, USA). Digital images were captured on a Leica DM4000 B LED microscope at a 5x magnification using a Leica DFC 450 camera and the Leica Application Suite V43 software (Leica Microsystems, Switzerland). All lighting conditions and magnifications were held constant and the investigator was unaware of the experimental groups. The color images were converted to 8-bit greyscale images. For each animal, the average optical density was determined at the following coordinates relative to bregma: -0.24 mm, -0.48 mm, -0.72 mm, and -0.96 mm, measuring a total area of 1,200,000 μm^2^. The measured values were corrected for non-specific background staining by subtracting values obtained from the cortex. The damage was calculated as the percent of the DAT-positive area in the lesioned striatum divided by the DAT-positive area of the untreated contralateral side.

### Statistical Analysis

All statistical analysis was performed using GraphPad Prism Version 6.0 (GraphPad Software, Inc). Differences between means were assessed, as appropriate, by two- or one-way ANOVA followed by Bonferroni *post hoc* analysis.

## Results

### 6-OHDA induces cell death in a time-dependent manner

It has been previously shown that activin A is able to protect against 6-OHDA-induced degeneration *in vitro* [6]. To investigate if activin A exerts the same neuroprotective effect *in vivo*, we first aimed to establish a time course of 6-OHDA-mediated cell death. Following unilateral injection with 6-OHDA, dopaminergic and total neuron numbers were quantified using stereological analysis of TH and NeuN positive cells, respectively, at 1, 2, and 3 weeks post lesioning.

No effect of direct physical damage caused by the infusion was found, with no significant differences in the number of TH positive cells between the unlesioned and “lesioned” sides of the sham injected animals (S1 Fig). Furthermore, no significant differences were found between sham and 6-OHDA injected animals on the unlesioned side, demonstrating that degeneration is confined to the ipsilateral side (S1 Fig). Analysis of NeuN positive cell numbers of the unlesioned side demonstrated similar results (S2 Fig).

**Fig 1:**
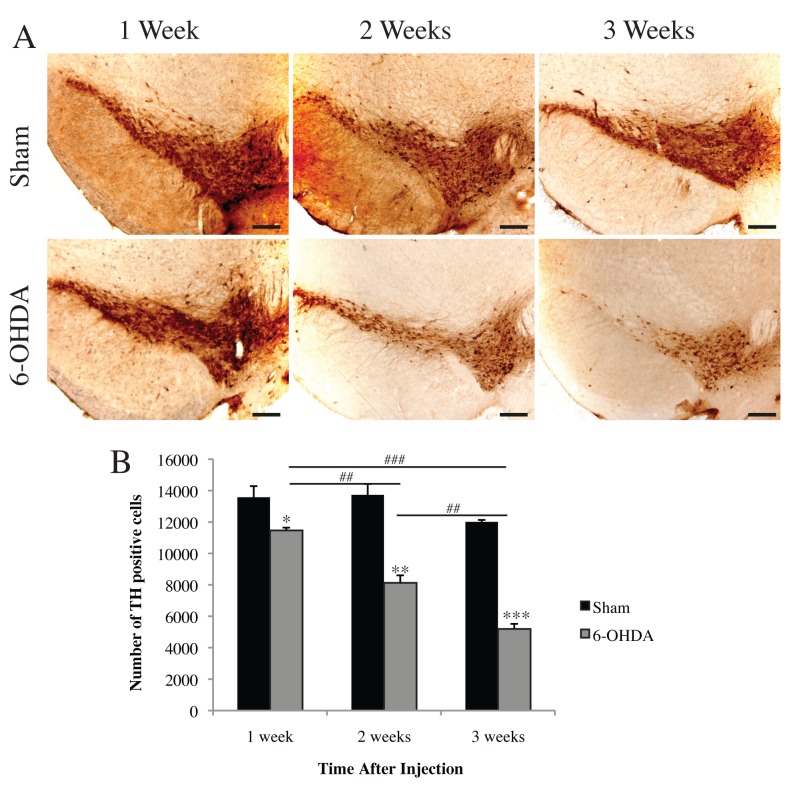
Time-dependent dopamine cell loss induced by 6-OHDA. (A) Representative images of TH-immunoreactive neurons in the SNpc. (B) Stereological quantification revealed a significant and progressive loss of dopaminergic cell numbers at 1, 2, and 3 weeks following unilateral injection of 6-OHDA or ascorbic acid (sham). All values represent the mean ± standard error of the mean (SEM) *p<0.05, **p<0.01, ***p<0.001 (compared to sham controls), ##*p*<0.01, ###*p*<0.001. N = 3 per group. Scale bar represents 200 μm.

Two-way ANOVA demonstrated a significant interaction between toxin and time on TH positive cell numbers in the SNpc (F_(2,12)_ = 13.10 *p*<0.001) indicating a significant effect of 6-OHDA on dopaminergic cell survival over time. Therefore separate one-way ANOVAs were conducted on toxin and time. Significant loss of TH positive cell numbers was observed at 1 (t_(1,4)_ = 2.889, *p*<0.05), 2 (t_(1,4)_ = 6.641 *p*<0.01), and 3 weeks (t_(1,4)_ = 19.65 *p*<0.001) following unilateral 6-OHDA lesioning compared to their sham controls (Fig 1A and 1B). A one-way ANOVA of 6-OHDA treatment indicated significant loss of TH positive cells with time (F_(2,6)_ = 80.30 *p*<0.001). A Bonferroni *post-hoc* analysis revealed a significant difference in TH positive cells between 1 and 2 weeks (*p*<0.01), 2 and 3 weeks (*p*<0.01), and 1 and 3 weeks post lesioning (*p*<0.001).

**Fig 2:**
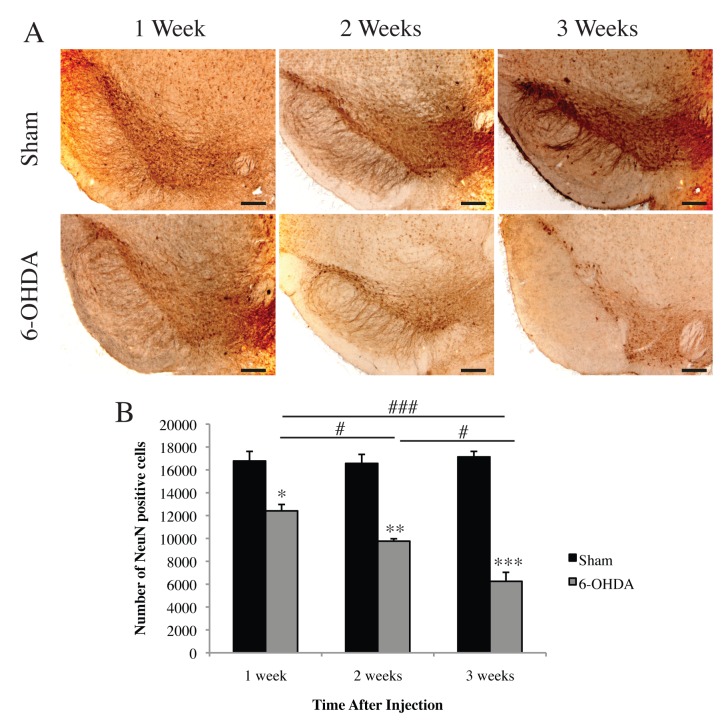
Time-dependent nigral cell loss induced by 6-OHDA. (A) Representative images of NeuN-immunoreactive neurons in the SNpc. (B) Stereological quantification revealed a significant and progressive loss of total cell numbers at 1, 2, and 3 weeks following unilateral injection of 6-OHDA or ascorbic acid (sham). All values represent the mean ± standard error of the mean (SEM) *p<0.05 **p<0.01, ***p<0.001 (compared to sham controls), #*p*<0.05, ###*p*<0.001. N = 3 per group. Scale bar represents 200 μm.

Two-way ANOVA of stereological quantification of total neuronal loss demonstrated a significant interaction between toxin and time on NeuN positive cell numbers in the SNpc (F_(2,12)_ = 12.96 *p*<0.01) indicating a significant effect of 6-OHDA on neuronal cell survival over time. Therefore separate one-way ANOVAs were conducted on toxin and time. Significant loss of NeuN positive cell numbers was observed at 1 (t_(1,4)_ = 4.325, *p*<0.05), 2 (t_(1,4)_ = 8.384 *p*<0.01), and 3 weeks (t_(1,4)_ = 11.86 *p*<0.001) following unilateral 6-OHDA lesion compared to their sham controls (Fig 2A and 2B). A one-way ANOVA of 6-OHDA treatment indicated significant loss of NeuN positive cells with time (F_(2,6)_ = 29.7 *p*<0.001). A Bonferroni *post-hoc* analysis revealed a significant difference in NeuN positive cells between 1 and 2 weeks (*p*<0.05), 2 and 3 weeks (*p*<0.05), and 1 and 3 weeks post lesioning (*p*<0.001). The results from this study demonstrate that unilateral 6-OHDA administration results in significant and progressive dopaminergic and total cell loss, with the greatest degeneration present at 3 weeks post-lesioning.

### Activin A increases survival of dopaminergic neurons against 6-OHDA-induced toxicity

**Fig 3:**
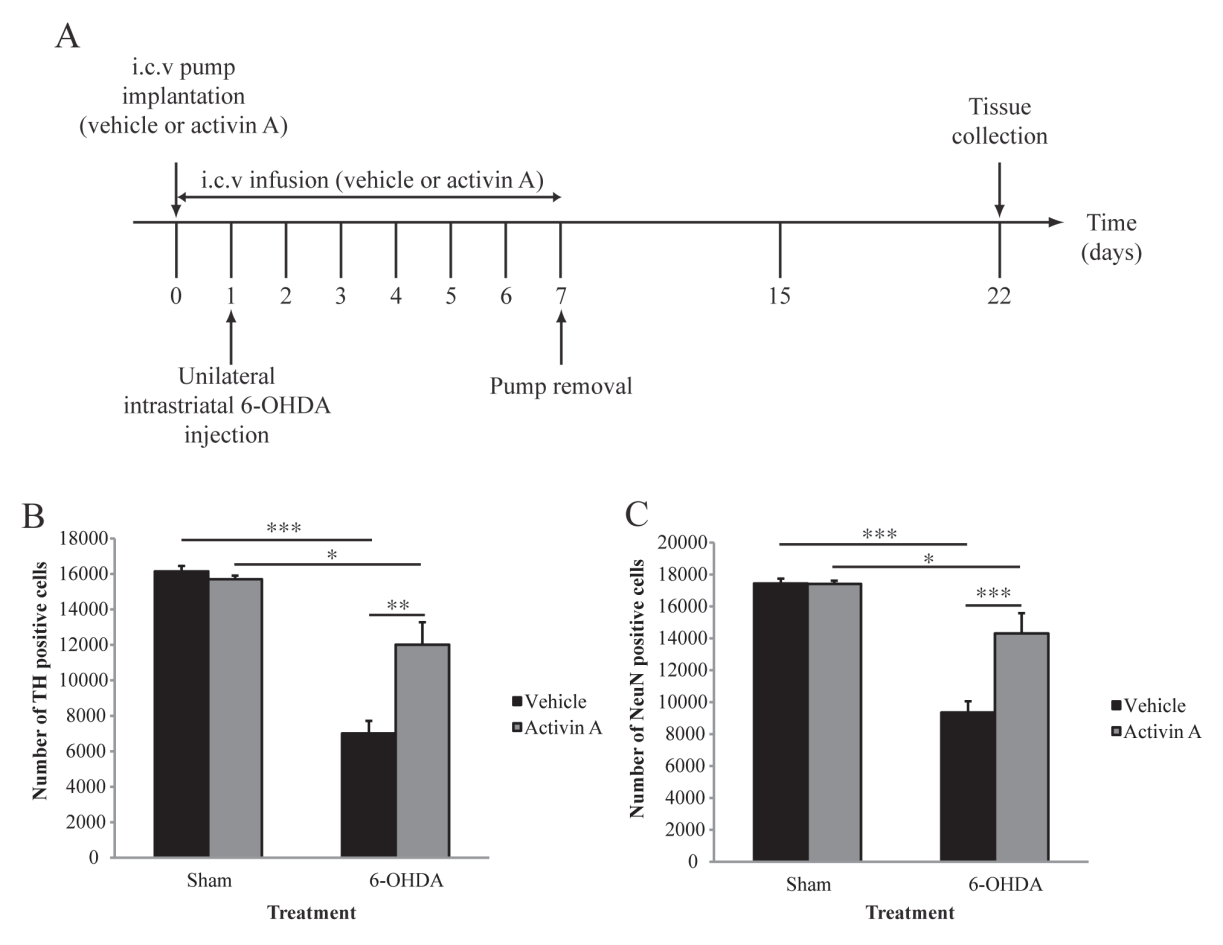
Activin A protects against 6-OHDA induced cell loss. (A) Experimental timeline detailing pump implantation, unilateral injection of 6-OHDA or ascorbic acid (sham), and tissue collection. (B) Stereological quantification of TH-immunoreactive neurons in SNpc demonstrates activin A protects dopaminergic neurons against 6-OHDA toxicity. (C) Stereological quantification of NeuN-immunoreactive neurons in SNpc demonstrates activin A protects total neuron numbers against 6-OHDA induced toxicity. All values represent the mean ± standard error of the mean (SEM). *p<0.05, **p<0.01, ***p<0.001. Sham + vehicle n = 4, sham + activin A n = 6, 6-OHDA + vehicle n = 7, 6-OHDA + activin A n = 7.

After establishing a time course of 6-OHDA-mediated cell death, in which counts at 3 weeks post lesioning revealed the highest level of cell loss in our study, we aimed to investigate if activin A is neuroprotective against 6-OHDA-induced degeneration. We therefore quantified the number of TH positive cells in the SNpc 3 weeks after 6-OHDA lesioning. Two-way ANOVA analysis of stereological quantification demonstrated a significant interaction between toxin and drug treatment (F_(1,20)_ = 9.288 *p*<0.01), suggesting an effect of activin A on lesion severity. Therefore separate one-way ANOVAs of toxin and drug treatment were conducted. Sham animals receiving activin A showed no significant differences in the number of TH positive cells compared to sham animals receiving vehicle (*p* = 0.2446), suggesting that activin A does not alter baseline levels of dopamine neurons (Fig 3A and 3B). As expected, the administration of 6-OHDA significantly decreased the number of dopaminergic neurons in both animals receiving the vehicle and activin A (*p*<0.001 and *p*<0.05, respectively). However, while 6-OHDA resulted in a significant decrease in TH positive cells in animals receiving activin A compared to their sham controls, there was a significant difference in dopaminergic neuron numbers between vehicle and activin A groups in 6-OHDA lesioned animals (Fig 3A and 3B *p*<0.01), suggesting a neuroprotective effect of activin A on dopaminergic cells against 6-OHDA-induced toxicity.

### Activin A increases survival of nigral neuronal populations against 6-OHDA-induced toxicity

While the use and quantification of TH as a marker for DA cell survival is a well established practice in animals models of PD research, it is necessary to control for the loss of this phenotypic marker in the absence of cell death. Therefore, to ensure that the neuroprotective effect of activin A in dopaminergic neurons against 6-OHDA-induced degeneration was not simply a result of decreased TH expression in vehicle treated animals, we also quantified the number of NeuN positive cells in the SNpc. Similarly to that seen with TH, two-way ANOVA demonstrated a significant interaction between the toxin and drug treatment (F_(1,20)_ = 13.12 *p*<0.01), suggesting an effect of activin A on 6-OHDA-induced cell loss. Therefore separate one-way ANOVAs of toxin and drug treatment were conducted. Sham animals receiving activin A showed no significant differences in the number of NeuN positive cells compared to sham animals receiving vehicle (*p* = 0.9286), suggesting that activin A does not alter baseline levels of total neuron numbers in the SNpc (Fig 3C). As expected, the administration of 6-OHDA significantly decreased total neuron numbers in both animals receiving vehicle and activin A (*p*<0.001 and *p*<0.05, respectively). However, while 6-OHDA resulted in a significant decrease in NeuN positive cells in animals receiving activin A compared to their sham controls, there was a significant difference in total neuron numbers between vehicle and activin A groups in 6-OHDA lesioned animals (Fig 3C *p*<0.001). As expected, the percentage of NeuN positive cells lost following 6-OHDA was very similar to that of TH positive cells from the same treatment group. Together, these results indicate that activin A protects total neuron numbers in the SNpc against 6-OHDA-induced toxicity.

### Activin A does not alter striatal dopamine levels following 6-OHDA

**Fig 4:**
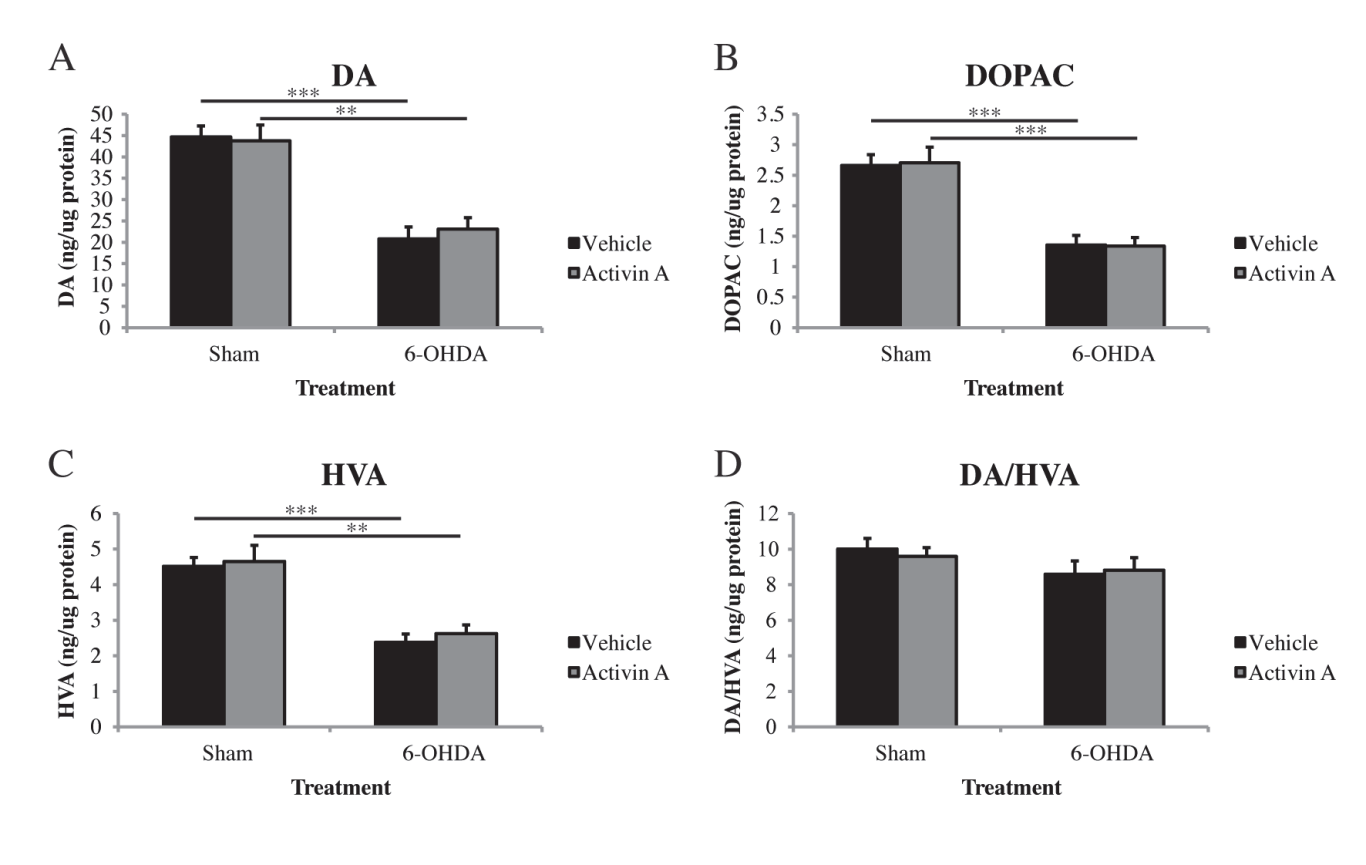
Activin A does not restore striatal catecholamine in 6-OHDA lesioned animals. HPLC-ECD quantification of striatal tissue revealed no significant effect of activin A on (A) DA, (B) DOPAC or (C) HVA levels following 6-OHDA compared to vehicle controls or the catabolism of DA to HVA (D). Sham = ascorbic acid. All values represent the mean ± standard error of the mean (SEM). *p<0.05, **p<0.01, ***p<0.001. Sham + vehicle n = 7, sham + activin A n = 7, 6-OHDA + vehicle n = 9, 6-OHDA + activin A n = 8.

The loss of dopaminergic neurons within the SN in PD results in a subsequent loss of DA in the striatum. As we demonstrated that activin A treatment is able to protect against both dopaminergic and total neuron loss induced by 6-OHDA toxicity, it would be reasonable to presume that activin A treatment would also result in a concurrent protection of striatal DA levels. We therefore quantified catecholamine levels from striatal tissue that was homogenized in solution containing 100 μM 5-HMeT (internal standard) and analyses for DA, DOPA, and HVA via HPLC coupled to an electrochemical detector was conducted. Two-way ANOVA analysis revealed a significant effect of toxin administration (F_(1,27)_ = 37.05 *p*<0.001), with both vehicle (*p*<0.001) and activin A (*p*<0.01) treated animals displaying significantly lower striatal DA levels compared to their sham controls. *Post hoc* analysis with Bonferroni corrections revealed no significant differences in DA levels in sham animals receiving vehicle or activin A (*p*>0.9999), indicating that activin A does not alter baseline levels of DA. However, in animals lesioned with 6-OHDA, the administration of activin A did not increase striatal DA levels in comparison to animals receiving vehicle (Fig 4A *p* = 0.6847), contrasting our previous neuroprotective effect of activin A in the SNpc.

Following its release from presynaptic terminals, DA either binds to and activates the DA receptors D1 to D5 [11], or is actively translocated from the extracellular space into presynaptic neurons and surrounding glial cells via the dopamine transporter (DAT) where it is then either repackaged into synaptic vesicles or degraded into its metabolites DOPAC and HVA [12]. We therefore quantified the amount of these metabolites within the striatum to determine if they were altered with the administration of activin A. Two-way ANOVA with *post hoc* Bonferroni corrections revealed no significant differences in DOPAC or HVA levels in sham animals receiving either vehicle or activin A (DOPAC *p*>0.9999; HVA *p*>0.9999) indicating that activin A does not alter baseline levels of these metabolites. While the administration of 6-OHDA significantly decreased both DOPAC and HVA levels in animals receiving vehicle (DOPAC *p*<0.001; HVA *p*<0.001) and activin A (DOPAC *p*<0.001; HVA *p*<0.01) compared to their sham controls, there was no significant difference in striatal DOPAC (*p*>0.9999) or HVA (*p* = 0.6829) levels in animals 6-OHDA lesioned animals receiving vehicle or activin A (Fig 4B and 4C). Furthermore, activin A does not alter the catabolism of DA to HVA, with two-way ANOVA analysis revealing no significant difference in the ratio of these two catecholamines between animals receiving vehicle or activin A and lesioned with 6-OHDA (Fig 4D *p*>0.9999).

### Striatal dopamine transporter expression is not restored with activin A administration

**Fig 5:**
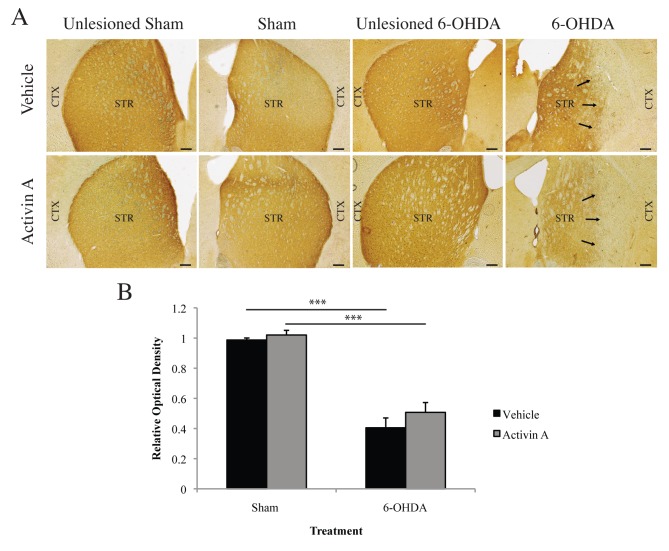
Activin A does not protect striatal DA terminals against 6-OHDA-induced toxicity. (A) Representative images of DAT-immunoreactive fibres in the striatum. Arrows represent areas of loss of DAT-positive fibres. (B) Optical density analysis demonstrates that activin A does not protect against the 6-OHDA-induced reduction in DAT-positive fibres in the striatum. Sham = ascorbic acid, CTX = cortex, STR = striatum. All values represent the mean ± standard error of the mean (SEM). ***p<0.001. N = 5 per group. Scale bar represents 200 μm.

In the striatum, the dopamine transporter (DAT) plays an important role for maintaining sufficient DA levels for release into the synaptic cleft, thus when striatal DAT loss reaches levels equivalent to those seen upon presentation of locomotor symptoms, a concomitant deficit in DA is produced [13–15]. To investigate if the inability of activin A to restore DA levels in the striatum following 6-OHDA, despite a protection of the DA-producing cells in the SNpc, was due to a deficit in DAT, we quantified immunohistochemical DAT expression in the striatum via densitometry. Two-way ANOVA with *post-hoc* Bonferroni corrections demonstrated no significant difference in DAT expression in sham animals receiving vehicle or activin A (*p*>0.9999), indicating that activin A does not alter baseline expression of DAT. While the administration of 6-OHDA resulted in a significant decrease of DAT expression in the striatum (vehicle *p*<0.001; activin A *p*<0.001), animals receiving activin A exhibited similar expression levels compared to their vehicle controls following lesioning with 6-OHDA (Fig 5A and 5B *p*>0.9999) suggesting that the neuroprotective effect of activin A may be confined to the SNpc.

## Discussion

Despite decades of preclinical and clinical research, the current pharmacological therapies for PD remain ineffective in the long term and are unable to halt ongoing nigrostriatal degeneration. However, the use of growth factors has received intense focus in recent years based on their ability to promote induction, specification, survival and maturation of developing neurons within the CNS. Since the discovery of GDNF in the early 90’s, it has been demonstrated that many DA neurotrophic factors are members of the TGFβ superfamily of proteins, making this family an attractive target for therapeutic intervention in PD.

Following promising neuroprotective and neurorestorative results in animal models, numerous clinical trials of growth factors in PD patients were conducted [16]. However, the results of these clinical trials proved largely disappointing, with some endpoints not met, site delivery and retrograde transport issues, and the presence of unwanted lesions dampening results [17–21]. Despite these translational issues, optimism remains that growth factors, and in particular those of the TGFβ superfamily, of which activin A is a member, will prove useful for PD.

In addition to its known neuroprotective effects in the hippocampus [2,4], it has been demonstrated that activin A attenuates degeneration induced by 6-OHDA [6] or MPP^+^ [5] administration *in vitro*, providing the first suggestion that activin A may exert neuroprotective effects in midbrain neurons. We therefore hypothesized that activin A would provide the same neuroprotective effects against 6-OHDA-induced degeneration *in vivo*. Unlike the MPTP model of PD, intrastriatal injections of 6-OHDA results in a delayed and protracted degenerative process, with changes in SNpc dendritic density, cell size, gene expression, and reduction in the number of TH positive cells occurring several days after the rapid loss of DA fibres surrounding the site of injection [22]. While a number of previous studies have investigated the time course of cell loss in the SNpc following 6-OHDA, many of these experiments were performed in rats [23–25] or focused on the early events of degeneration [22]. We therefore established a time course of degeneration in drug naïve mice to determine the point at which midbrain neuronal loss is greatest. Stereological estimations of TH and NeuN positive cells in the SNpc demonstrated that unilateral striatal 6-OHDA administration resulted in a time-dependent loss of both dopaminergic and total neuron populations, with cell loss present at 1 week and showing constant progression up to the end of the study at 3 weeks post lesioning.

These findings raise important considerations regarding the timing and delivery of therapeutic agents following intrastriatal administration of 6-OHDA. Indeed, numerous studies have demonstrated that the timing and site of administration of GDNF relative to the lesion can modulate the neuroprotective effects of the trophic factor against 6-OHDA toxicity [26–29]. In the intrastriatal 6-OHDA lesion model, prior GDNF administration is able to almost completely rescue the number of nigral DA cell bodies, stimulate axonal sprouting and regrowth, and stimulate DA turnover and function. However when administered after 6-OHDA, GDNF is unable to provide the same neuroprotective effects, with a significant DA denervation still occurring within the striatum [28,29]. We therefore administered activin A for a full 7 days, beginning the day prior to injection of 6-OHDA, and quantified the number of remaining TH and NeuN positive cells 3 weeks after 6-OHDA administration. Stereological analysis revealed that exogenous activin A resulted in significant protection of both dopaminergic and total neuron numbers in the SNpc against 6-OHDA toxicity. This pronounced neuroprotective effect of intracerebroventricular activin A may result from either activin A inhibiting the initial degenerative phase of 6-OHDA and thus preventing further cell loss, or exerting neurotrophic effects past the time of removal of the osmotic pump.

The loss of DA producing cells within the SN of PD patients results in a subsequent loss of DA within the striatum, and ultimately a disruption of the finely tuned signaling of the basal ganglia. Our studies reveal that activin A can significantly increase the survival of midbrain dopaminergic neurons following intrastriatal 6-OHDA administration and it could therefore be hypothesized that activin A would also result in a subsequent protection of striatal DA levels. HPLC-ECD analysis of catecholamine levels in the striatum of vehicle and activin A treated animals revealed that while 6-OHDA resulted in a significant loss of striatal DA, animals receiving activin A were not significantly different from vehicle controls in DA levels or its metabolites DOPAC and HVA. Furthermore this was not due to activin A altering the catabolism of DA, with no difference in the ratio of DA to HVA found between animals receiving vehicle or activin A. These results suggest that while activin A protects 6-OHDA-induced degeneration of the DA producing nigral cell bodies, this neuroprotection does not translate to a subsequent protection of DA levels in the striatum.

In the striatum, the dopamine transporter plays an important role for maintaining sufficient DA levels for release into the synaptic cleft, thus when striatal DAT loss reaches levels equivalent to those seen upon presentation of locomotor symptoms, a concomitant deficit in DA is produced [13–15]. It could therefore be hypothesized that the inability of activin A to restore DA levels, despite the increase in survival of DA producing cells, is due to the degeneration in the striatum of the projecting fibres from the nigral cell bodies. Quantification of immunohistochemical DAT expression in the striatum demonstrated that activin A was unable to protect striatal DA terminals from degeneration, with DAT expression unchanged between vehicle and activin A treated animals following 6-OHDA. This may explain the lack of DA restoration and would suggest that activin A is unable to maintain striatal integrity following 6-OHDA induced toxicity.

The results of this study raise a number of intriguing questions. In particular, what is the reason for the selective neuroprotection of the nigral cells? Numerous studies have demonstrated that the proximity to the site of lesion increases the neuroprotective effect of trophic factors such as GDNF and Neurturin in animal models [26,30–34]. It is therefore surprising that the neuroprotective effect of activin A appears to be confined to the cell bodies in the SNpc with no effect on the DA terminals and DA levels, given the location of activin A administration is closer to the striatum relative to the SN. It may also be the case that the site of delivery of activin A affects its actions more generally. Alternatively, it is possible that the lack of effect of activin A in the striatum, in our study, may result from differing direct and/or indirect actions on the soma vs. the projecting fibres of the dopaminergic system. Furthermore, investigations in other animal models of PD may provide the basis of determining the neuroprotective mechanisms of activin A based on the mechanisms of degeneration of the model used. These questions raised by our study are important not only to further investigations of activin A, but also to the investigation of growth factors in PD more generally.

With the introduction of L-Dopa as a PD therapy approximately 50 years ago, the outlook and quality of life for patients dramatically changed, however there still remains no therapy that treats the underlying degeneration that is characteristic of the disease. These findings provide the first evidence that exogenous activin A is able to significantly increase the survival of midbrain dopaminergic and total neuron populations following intrastriatal 6-OHDA administration.

## Supporting Information

S1 Datasets Datasets for figures. (XLSX)

S1 Fig 6-OHDA-induced loss of dopaminergic neurons is confined to the ipsilateral side. (A) Representative images of TH-immunoreactive neurons in the SNpc. (B) Stereological quantification revealed no significant loss of dopaminergic cell numbers of the unlesioned hemisphere at 1, 2, and 3 weeks in ascorbic acid (sham) animals. (C) No significant loss of dopaminergic cell numbers was found between sham and 6-OHDA injected animals in the unlesioned hemisphere. All values represent the mean ± standard error of the mean (SEM). N = 3 per group. Scale bar represents 200μm.
(TIF)

S2 Fig 6-OHDA-induced loss of total nigral neurons is confined to the ipsilateral side. (A) Representative images of NeuN-immunoreactive neurons in the SNpc. (B) Stereological quantification revealed no significant loss of total cell numbers of the unlesioned hemisphere at 1, 2, and 3 weeks in ascorbic acid (sham) animals. (C) No significant loss of total cell numbers was found between sham and 6-OHDA injected animals in the unlesioned hemisphere. All values represent the mean ± standard error of the mean (SEM). N = 3 per group. Scale bar represents 200μm.
(TIF)
