# Supplementary figures and images for: Activin A Protects Midbrain Neurons in the 6-Hydroxydopamine Mouse Model of Parkinson’s Disease
Source: PLoS One. 2015 Apr 22;10(4):e0124325. doi: 10.1371/journal.pone.0124325 (PMC4406584; doi:10.1371/journal.pone.0124325)

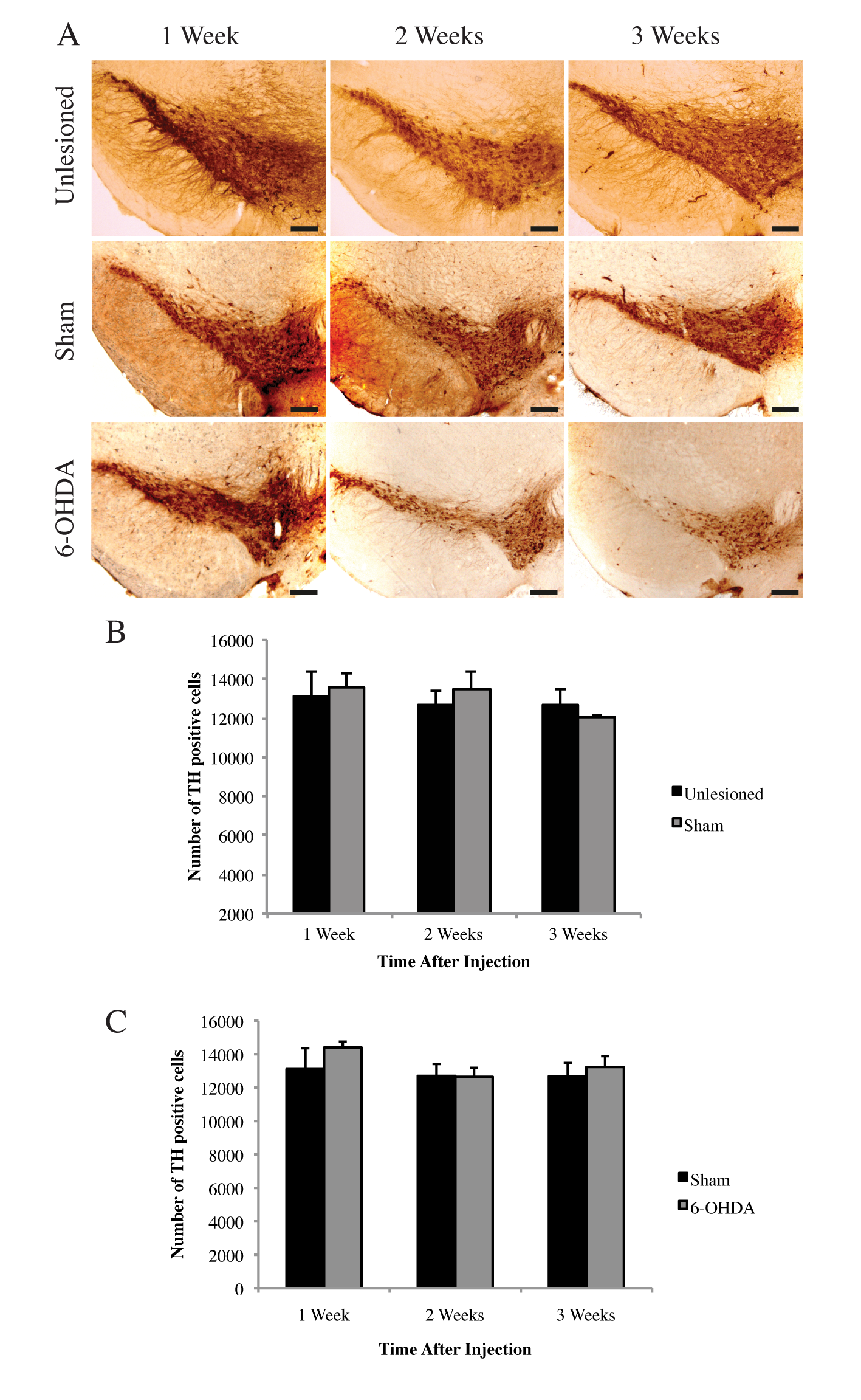

Supplement: S1 Fig — (A) Representative images of TH-immunoreactive neurons in the SNpc. (B) Stereological quantification revealed no significant loss of dopaminergic cell numbers of the unlesioned hemisphere at 1, 2, and 3 weeks in ascorbic acid (sham) animals. (C) No significant loss of dopaminergic cell numbers was found between sham and 6-OHDA injected animals in the unlesioned hemisphere. All values represent the mean ± standard error of the mean (SEM). N = 3 per group. Scale bar represents 200μm. (TIF) [file pone.0124325.s002.tif]

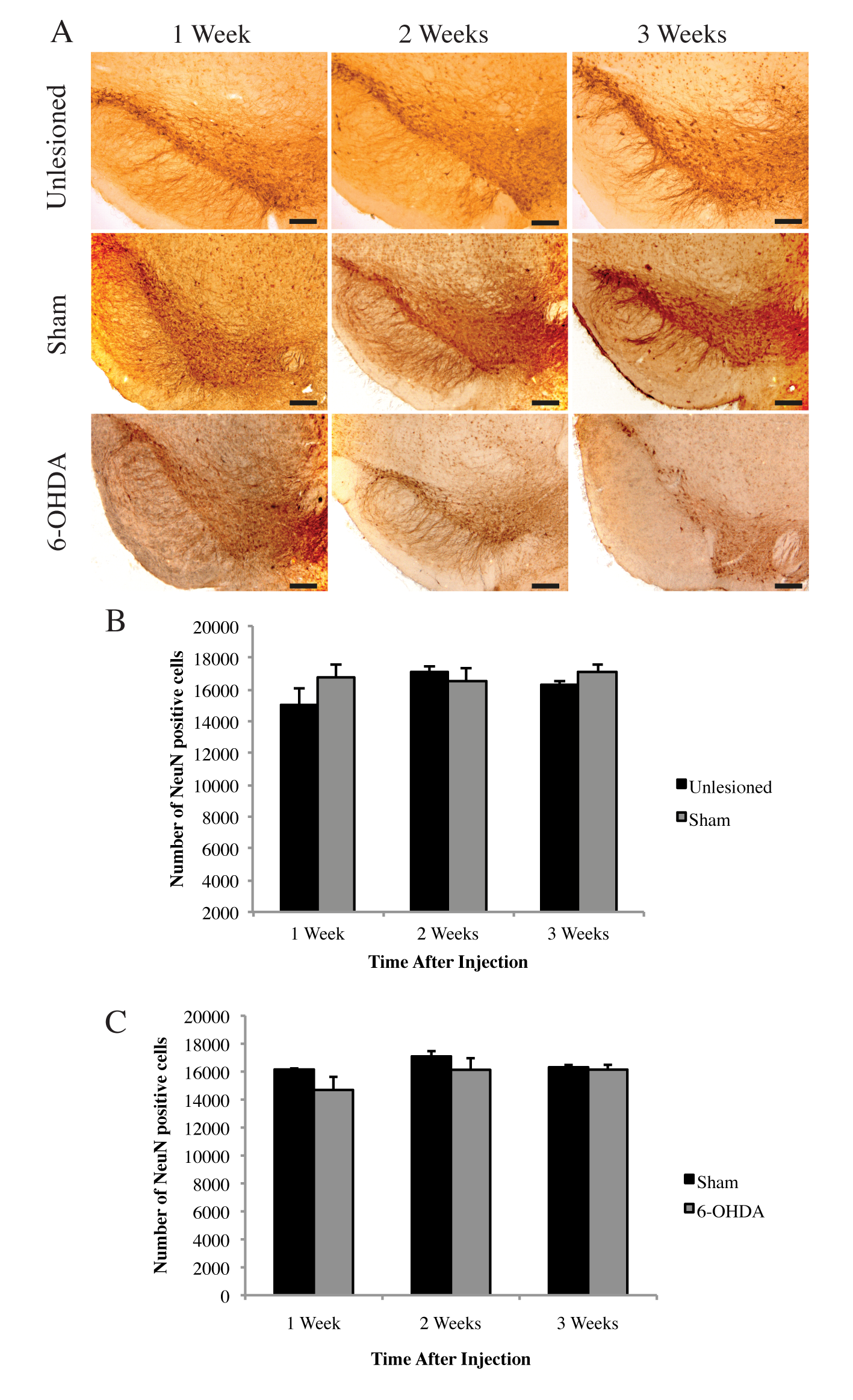

Supplement: S2 Fig — (A) Representative images of NeuN-immunoreactive neurons in the SNpc. (B) Stereological quantification revealed no significant loss of total cell numbers of the unlesioned hemisphere at 1, 2, and 3 weeks in ascorbic acid (sham) animals. (C) No significant loss of total cell numbers was found between sham and 6-OHDA injected animals in the unlesioned hemisphere. All values represent the mean ± standard error of the mean (SEM). N = 3 per group. Scale bar represents 200μm. (TIF) [file pone.0124325.s003.tif]
